# Identification of Novel Potential VEGFR-2 Inhibitors Using a Combination of Computational Methods for Drug Discovery

**DOI:** 10.3390/life11101070

**Published:** 2021-10-11

**Authors:** Mohammad M. Al-Sanea, Garri Chilingaryan, Narek Abelyan, Arsen Sargsyan, Sargis Hovhannisyan, Hayk Gasparyan, Smbat Gevorgyan, Sarah Albogami, Mohammed M. Ghoneim, Ahmed K. Farag, Ahmed A. B. Mohamed, Ashraf K. El-Damasy

**Affiliations:** 1 Pharmaceutical Chemistry Department, College of Pharmacy, Jouf University, Sakaka 72341, Saudi Arabia; mmalsanea@ju.edu.sa; 2 Institute of Molecular Biology of NAS RA, Yerevan 0014, Armenia; a_sargsyan@mb.sci.am; 3 Institute of Biomedicine and Pharmacy, Russian-Armenian University, Yerevan 0051, Armenia; narek.abelyan@rau.am; 4 Foundation for Armenian Science and Technology, Yerevan 0033, Armenia; smbatg@fast.foundation; 5 Department of Mathematics and Mechanics, Yerevan State University, Yerevan 0025, Armenia; saqohovhannisyan0199@gmail.com (S.H.); haykgasparyan012@gmail.com (H.G.); 6 Armenian Bioinformatics Institute, Yerevan 0014, Armenia; 7 Department of Biotechnology, College of Science, Taif University, Taif 21944, Saudi Arabia; dr.sarah@tu.edu.sa; 8 Department of Pharmacy Practice, College of Pharmacy, Al Maarefa University, Ad Diriyah 13713, Saudi Arabia; mghoneim@mcst.edu.sa; 9 Curachem Inc., Cheongju-si 28161, Korea; akm190@gmail.com; 10 Department of Medicinal Chemistry, Faculty of Pharmacy, Mansoura University, Mansoura 35516, Egypt; ahmed_smt@yahoo.com (A.A.B.M.); ashraf.el-damasy@kist.re.kr (A.K.E.-D.); 11 Brain Science Institute, Korea Institute of Science and Technology (KIST), Seoul 02792, Korea

**Keywords:** VEGFR2, tivozanib, virtual screening, drug discovery, molecular dynamics simulations

## Abstract

The vascular endothelial growth factor receptor 2 (VEGFR-2) is largely recognized as a potent therapeutic molecular target for the development of angiogenesis-related tumor treatment. Tumor growth, metastasis and multidrug resistance highly depends on the angiogenesis and drug discovery of the potential small molecules targeting VEGFR-2, with the potential anti-angiogenic activity being of high interest to anti-cancer research. Multiple small molecule inhibitors of the VEGFR-2 are approved for the treatment of different type of cancers, with one of the most recent, tivozanib, being approved by the FDA for the treatment of relapsed or refractory advanced renal cell carcinoma (RCC). However, the endogenous and acquired resistance of the protein, toxicity of compounds and wide range of side effects still remain critical issues, which lead to the short-term clinical effects and failure of antiangiogenic drugs. We applied a combination of computational methods and approaches for drug design and discovery with the goal of finding novel, potential and small molecule inhibitors of VEGFR2, as alternatives to the known inhibitors’ chemical scaffolds and components. From studying several of these compounds, the derivatives of pyrido[1,2-a]pyrimidin-4-one and isoindoline-1,3-dione in particular were identified.

## 1. Introduction

The kinase insert domain receptor (KDR, a type IV receptor tyrosine kinase), also known as vascular endothelial growth factor receptor 2 (VEGFR-2), is largely recognized as a potent therapeutic molecular target for the development of angiogenesis-related tumor treatment [1]. The binding of VEGF to the VEGFR induces conformational changes, in particular the exposure of the ATP binding site and the subsequent dimerization of VEGFR [2]. The autophosphorylation or dephosphorylation of specific tyrosine residues of VEGFR leads to the activation of several downstream signal transduction pathways, such as the p38-MAPK, Raf/ MEK/ERK and PI3K/PKB pathways [1,2]. The activations of these molecular pathways mediate cell proliferation, increase cell vascular permeability and, ultimately, lead to angiogenesis [3,4]. VEGFR-2 consists of three structural domains: the extracellular ligand-binding domain, the transmembrane domain, and the tyrosine kinase domain. VEGFR-2 inhibitors interact with the ATP-binding site in the catalytic domain of the receptor and prevent its dimerization and autophosphorylation processes [5]. In recent decades, several monoclonal antibodies, such as bevacizumab [6], ramucirumab [7], and aflibercept [8], as well as multiple small molecule inhibitors of the VEGFR-2, such as sorafenib [9], sunitinib [10], pazopanib [11], vandetanib [12], axitinib [13], regorafenib [14], nintenanib [15], lenvatinib [16] and apatinib [17] were approved for the treatment of different type of cancers, including metastatic colorectal cancer renal carcinoma, non-small cell lung cancer, and thyroid cancer, etc. The fact that the growth, metastasis and multidrug resistance of tumors is highly dependent on the angiogenesis [2] and since VEGFR-2 is one of the key receptors which regulates angiogenic function, the drug discovery of potential small molecules targeting VEGFR-2 with potential anti-angiogenic activity is of high interest in anti-cancer research. Despite the fact that multiple VEGFR-2 inhibitors have already been discovered, the endogenous and acquired resistance of the protein and the wide range of side effects of existing drug compounds still remain critical issues leading to short-term clinical effects and failure of antiangiogenic drugs [18,19]. These issues are associated with the fact that, due to the high structural similarity of VEGFR proteins family, VEGFR2 binding small molecules lack selectivity and can interact with all of the other VEGF family receptors. Moreover, VEGFR-2′s catalytic domain where interaction with the small molecule inhibitors occurs, has a high structural similarity to the other tyrosine kinase receptors, such as the platelet-derived growth factor receptors (PDGFRs), and the colony-stimulating factor 1 receptor (CSF1R), as well as fms such as tyrosine kinase 3 (FLT-3), c-Kit, etc., that can cause unpredicted reactions from side effects. VEGFR-2 small molecule inhibitors are generally classified into two types: type 1 (DFG-in) and type 2 (DFG-out). The type 2 inhibitors, such as sorafenib, lenvatinib, apatinib and tivozanib bind in the hinge region and one of the hydrophobicity regions (HYD-II), which is also referred to as an allosteric site. Type 2 inhibitors are regarded as indirect ATP competitive inhibitors and can interact with other amino acid residues of the binding site, which provides the opportunity to raise the selectivity of the small molecule inhibitors toward the VEGFR-2 protein. This study was devoted to the application of the combination of modern computer-aided drug design and discovery methods and approaches, such as ligand- and structure-based virtual screening, molecular dynamics simulations, binding free energy calculations and a chemical similarity analysis. The goal here is to find novel and promising VEGFR-2-binding small molecules with a potential anti-angiogenic activity.

## 2. Materials and Methods

The dataset of purchasable compounds from ZINC20 [20] chemical database for ligand discovery (http://zinc20.docking.org/) was downloaded and filtered using the PAINS filter [21]. ROCS [22] 3.4.1.0. (OpenEye Scientific Software, Santa Fe, NM, www.eyesopen.com, accessed on 10 September 2021) was used for ligand-based virtual screening and initial filtering of the dataset for compounds based on their shape similarity to the tivozanib (reference ligand). Two scores of ROCS software were used to rank compounds in comparison to the reference ligand. “Shape Tanimoto” score was used to rank molecules against the query molecule based on their shape similarity and Color Tanimoto score, which counted appropriate overlap of groups which describe properties, such as H-bond donors and acceptors, cations, anions, rings, etc.

Molecular docking of potential VEGFR-2 binding compounds retrieved from previous stages was performed using the ICM-PRO [23] v. 3.9-2b software (MolSoft L.L.C., www.molsoft.com, accessed on 10 September 2021), that showed high performance and accuracy among both academic and commercial software based on benchmark studies for molecular docking and virtual screening software [24,25]. ICM-PRO software used docking algorithm that was based on the Monte Carlo minimization approach and scoring function and was a weighted sum of multiple parameters, including van der Waals energy of the ligand-target interactions, internal force field energy of the ligand, hydrogen bonding interactions, hydrogen bond donor–acceptor desolvation energy and hydrophobic free energy gain, among others [23,26]. Crystal structure of VEGFR2 protein in complex with tivozanib (PDB ID: 4ASE [27], resolution:1.83 Å) was downloaded from Protein Data Bank [28] and used for structure-based virtual screening. Tivozanib, which was a potent, selective inhibitor of VEGFRs and was recently approved by the FDA for the treatment of adult patients with relapsed or refractory advanced renal cell carcinoma (RCC) [29], was used as a reference ligand for virtual screening and molecular dynamics simulations. 

Molecular dynamics simulations of selected compounds and the reference ligand were performed using AMBER20 [30] software package. The ff14SB force field [31] and General Amber Force Field [32] (GAFF) with AM1-BCC [33] charge model were used for parameterization of protein and chemical compounds, respectively. Complexes of VEGFR-2 with docked selected compounds and reference ligand, obtained from the previous stage, were used as starting positions for corresponding molecular dynamics simulations. Used protein-ligand complexes were solvated in TIP3P water model and Na+/Cl− ions at 150 mM concentration [34]. The Monte Carlo barostat [35] and Langevin thermostat [36] were used to keep the temperature at 310.15 K. The Particle Mesh Ewald [37] (PME) method was used for the long-range electrostatic interactions. Bonds involving hydrogen were constrained using the SHAKE algorithm [38]. All variable parameters for aforementioned methods were set in accordance with our similar, previous study [39]. The first 5 ns of each simulation covered minimization and equilibration stages, while the last 100 ns covered conventional molecular dynamics simulation. Binding free energy calculations of the interactions between the studied compounds and VEGFR2 were performed using molecular mechanics, Poisson–Boltzmann or generalized Born and surface area continuum solvation (MM/PBSA and MM/GBSA) methods [40]. MMPBSA.py [41] program was used to perform binding free energy calculations. For every simulation binding free energy calculation performed, 250 snapshots were collected from the last 20 ns of trajectory with equal intervals. 

Clusterization dendrogram was obtained using ICM-PRO software (detailed description of used methods can be found through the following link: www.molsoft.com/gui/clustering, accessed on 10 September 2021). The method for generating hashed chemical fingerprints (MolSoft’s Fingerprints) developed by MolSoft was used as a molecular descriptor for clustering. The unweighted pair group method using averages (UPGMA), which was a agglomerative (bottom-up) hierarchical clustering method recommended by MolSoft’s developers, was used for clusterization. Docked complexes were visualized using PyMol [42]. Comparative analysis of physicochemical features of the selected compounds with reference ligand (tivozanib) was performed using OpenEye ROCS’s ROCSReport utility, where, for the 2D Graph Similarity Score, the pink color highlights parts of the hit molecule that are dissimilar to the reference ligand, while the “yellow to dark green” color gradient highlights the similarity of the bonds.

## 3. Results 

**Figure 1:**
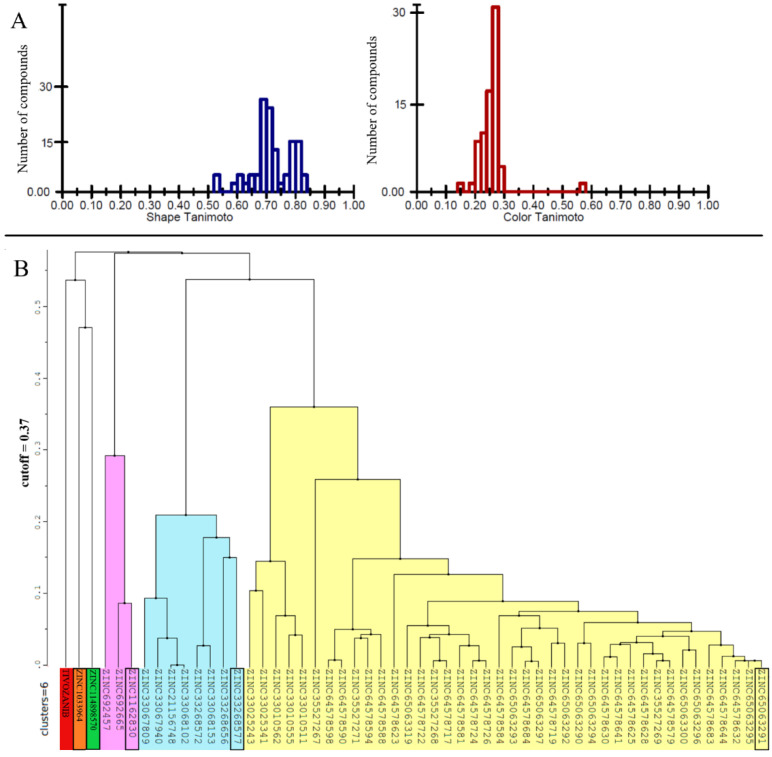
(**A**) Similarity analysis of the identified 53 chemical compounds to the co-crystallized ligand (tivozanib). (**B**) Clusterization of the 53 identified compounds with higher docking scores in comparison to tivozanib. For each cluster, ZINC ID of the representative compound with highest docking score is highlighted in black.

Five hundred thousand compounds with the highest shape similarity to the reference ligand, tivozanib, were retrieved from the ZINC’s dataset of purchasable compounds using OpenEye’s ROCS tool. The molecular docking of the selected 500,000 compounds against the ATP-binding site of the VEGFR2 protein (PDB ID: 4ASE) was performed using ICM-PRO software. Fifty-three compounds with higher or closer docking scores, when compared to the tivozanib (Table S1), were identified as a result of the molecular docking. A similarity analysis comparing the identified compounds with the tivozanib (Figure 1A) demonstrated that some of the compounds had a relatively close shape similarity to the reference ligand (Shape Tanimoto score > 0.8, while almost all of them had different chemical scaffolds and functional groups compared to the reference ligand (Color Tanimoto < 0.3). Based on their structural similarity, these fifty-three compounds could be divided into six clusters (Figure 1B).

**Figure 2:**
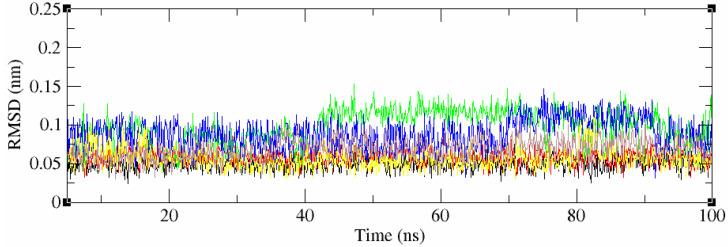
RMSD values of the 5 studied compounds and co-crystalized ligand. Black – tivozanib, red—ZINC114898570, green—ZINC1162830, blue—ZINC1033964, yellow—ZINC33268577, brown—ZINC65063291.

The 2D structures of all 53 compounds and tivozanib are presented in Table S2. It is noteworthy that one of the identified compounds, ZINC114898570 (fruquintinib), was a potent and highly selective small molecule inhibitor of VEGFRs, including the VEGFR2 protein, approved in China for use in the treatment of metastatic colorectal cancer (CRC) and currently under Fast Track designation in the FDA for the USA. This is also a good indicator of the validity of the computational methods and approaches used. The chemical compounds with the highest docking scores within the cluster, and tivozanib, were selected for subsequent MD simulations in order to validate the predicted binding poses and study stability of their interactions with the VEGFR2 protein, and to calculate the binding free energies of these interactions. All five of the studied compounds demonstrated a high stability during the 100 ns of molecular dynamics simulations which were performed (RMSD < 0.1 Å, Figure 2). However, tivozanib, ZINC114898570 and ZINC33268577 demonstrated exceptional stability during the performed simulations which could be seen from the RMS deviation plots. 

**Figure 3:**
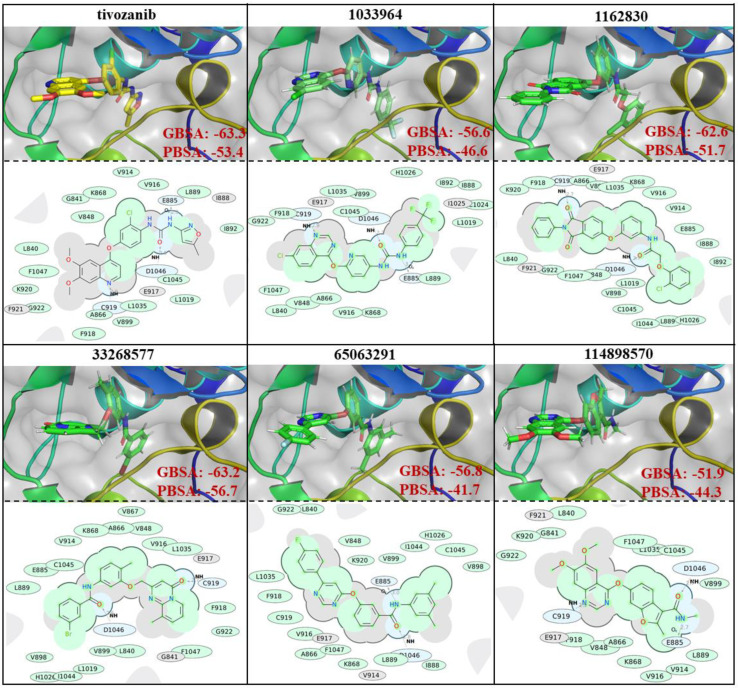
Binding poses, 2D interaction diagrams and interaction binding free energies (GBSA and PBSA, presented values are in kcal/mols) of tivozanib and 5 selected compounds.

The similarities and differences in the binding poses of the studied compounds within the ATP-binding site of the VEGFR2 protein can be seen in Figure 3. The binding free energy calculations, based on the performed simulations, showed that all the selected compounds demonstrated a strong binding affinity which was close to the reference ligand. Tivozanib demonstrated a free binding energy of −63.3 and −53.4 kcal/mol based on the GBSA and PBSA calculations, respectively. The compounds, ZINC1162830 and ZINC33268577, showed similar values of binding free energies to tivozanib, GBSA and PBSA. Based on the PBSA calculations, the binding free energy of the interaction between ZINC33268577 and VEGFR2 slightly exceeded that of tivozanib. The other three studied compounds demonstrated slightly weaker values of binding free energy compared to the aforementioned compounds, based on both PBSA and GBSA calculations. The ligand efficiency (LE) was another useful metric that helped to indicate molecules with optimal combinations of physicochemical properties and was essentially the ratio of the binding free energy to the number of the non-hydrogen atoms of compound. LE were calculated for six selected compounds based on their PBSA values (as a relatively more reliable binding free energy calculation method), and according to the obtained LE values, the compounds ranked in the following order (from best to worst): ZINC33268577 (LE: −1.84), tivozanib (LE: −1.66), ZINC114898570 (fruquintinib, LE: −1.52), ZINC1033964 (LE: −1.47), ZINC1162830 (LE: −1.44) and ZINC65063291 (LE: −1.35).

Based on the obtained 2D interaction diagrams, ZINC1033964 and ZINC114898570, along with the reference compound, tivozanib, all demonstrated the formation of three hydrogen bonds with E885, C919 and D1046, as well as the amino acid residues of the ATP-binding site of VEGFR2 protein (Figure 3). ZINC1162830 and ZINC33268577 showed a hydrogen bond formation only with the D1046 and C919 residues, while ZINC65063291 showed the formation of a hydrogen bond with E885 and D1046. 

Based on the obtained results, the V916, L889, L1035, A866, F918, G922, F1047, L840, V848 and K868 amino acid residues of the ATP-binding domain of the VEGFR2 protein are important for hydrophobic interactions, since they are common interacting residues with all the studied compounds. The C1045 and V899 amino acid residues are also of a higher importance for hydrophobic interactions with the small molecules, since interactions with these residues were observed in five out of six of the studied compounds (Figure 3). All of the studied compounds, except ZINC114898570 and tivozanib, showed additional interactions with H1026. ZINC1162830, ZINC33268577 and ZINC65063291 showed additional interactions with V898 and I1044. ZINC1033964, ZINC1162830 and ZINC65063291 have additional interaction with I888. Each of following compounds: ZINC1033964, ZINC1162830 and ZINC33268577 each showed a unique hydrophobic interaction with only one amino acid residue: C1024, V889 and V867, respectively. 

**Figure 4:**
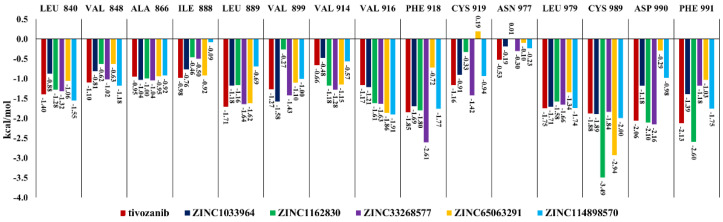
The common interacting amino acid residues of the ATP-binding site of VEGFR2 and their contribution to the interaction with the studied compounds and tivozanib in terms of binding energy.

The most commonly interacting amino acid residues of the VEGFR2 ATP-binding site among tivozanib and the five studied compounds, based on their contributions to the binding free energies, are presented in Figure 4. Tivozanib, ZINC1162830 and ZINC33268577 have stronger interactions in terms of the binding free energies within the amino acid residues of the VEGFR2 ATP-binding site, compared to other three studied compounds. Among these three compounds, ZINC33268577 has an exceptionally strong interaction with the PHE 918 and a relatively strong interaction with CYS 919. ZINC1162830 has an exceptionally strong interaction with the CYS 989 and a relatively strong interaction with PHE 991. Overall, compound ZINC33268577 demonstrated a strong interaction with more amino acid residues within the ATP-binding site of VEGFR2 compared to the other studied compounds (Figure 4).

**Figure 5:**
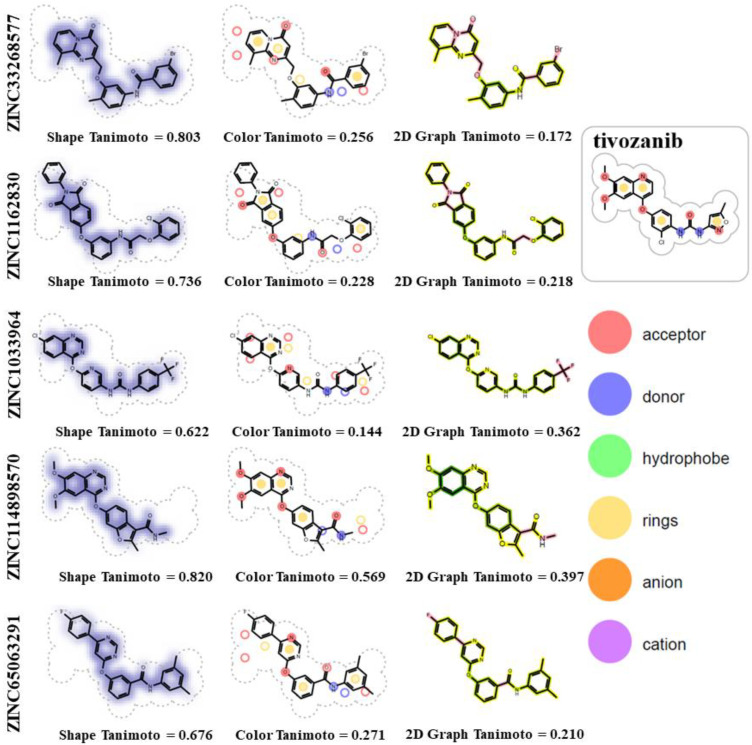
Chemical similarity analysis of ZINC33268577 and ZINC1162830 with reference ligand (tivozanib).

The identified compounds, in particular ZINC33268577 (3-bromo-N-[4-methyl-3-[(9-methyl-4-oxopyrido [1,2-a]pyrimidin-2-yl)methoxy]phenyl]benzamide) and ZINC1162830 (2-(2-chlorophenoxy)-N-[3-(1,3-dioxo-2-phenylisoindol-5-yl)oxyphenyl]acetamide) are similar to tivozanib in terms of the molecular shape of the compounds (Figure 5, Shape Tanimoto values of 0.803 and 0.736) and, at the same time, have different chemical groups (Figure 5, Color Tanimoto values of 0.256 and 0.218). Both of these compounds have one H-bond donor atom and five H-bond acceptor atoms, while tivozanib has two H-bond donors and seven H-bond acceptors. ZINC33268577, tivozanib and ZINC1162830 have 5, 6 and 7 rotatable bonds, respectively.

## 4. Discussion

The currently known VEGFR2 inhibitors, including the ones in the preclinical and clinical stages, already cover a relatively wide chemical space. VEGFR2 inhibitors include derivatives of quinazolines, quinolines (such as tivozanib), oxazolpyrrolocarbazoles, oxazoles phthalazines, furopyrazines, imidazolins, pyrrolotriazines, pyrrolopyrimidines, oxindoles, isothiazoles and others. Significant efforts have been made in the R&D of VEGFR2 inhibitors regarding anti-angiogenic activity, with several compounds being approved as drugs. However, the endogenous and acquired resistance of the protein, toxicity of compounds and wide range of side effects still remain critical issues, leading to the short-term clinical effects and failure of antiangiogenic drugs. We applied a combination of computational approaches for drug design and discovery with the goal of finding novel potential VEGFR2 inhibitors with an anti-angiogenic activity. As the result of the study, several compounds with interesting chemical scaffolds were identified. Two compounds in particular, ZINC33268577 and ZINC1162830, which were derivatives of pyrido[1,2-a]pyrimidin-4-one and isoindoline-1,3-dione, respectively, demonstrated a potency as VEGFR2 inhibitors, in comparison to tivozanib, which was recently approved by the FDA for the treatment of adult patients with relapsed or refractory advanced renal cell carcinoma (RCC) and was a potent, selective inhibitor of VEGFRs, including VEGFR2. The identified compounds shared chemical similarities to tivozanib and other known VEGFR2 inhibitors, but included chemical components that were not previously studied in depth. ZINC1033964 had a quinazolin chemical group, similarly to some known drugs, such as ZINC114898570 (fruquintinib) and vandetanib. ZINC6506329 and ZINC1033964 contained the pyridine group, which occured in many VEGFR2 inhibitors, such as sorafenib and apatinib. ZINC1033964 contained the phenyl urea group which could be found in many VEGFR2 inhibitors, including sorafenib, levantinib and tivozanib. Some of the approved drugs and compounds that are currently under investigation contain indole/indoline chemical groups, while one of the identified compounds, ZINC1162830, contains isoindole chemical group. It was noteworthy that all of the selected compounds contained the carboxamide group, which was a part of many drugs, including the aforementioned approved drugs and also drugs such as ponatinib, carbozantinib, sunitinib and others. These compounds demonstrated a stable interaction and low binding energy, and were similar to the tivozanib interaction patterns with the amino acid residues of the ATP-binding site of VEGFR2. Typical type 2 small molecule inhibitors of VEGFR2, such as sorafenib, lenvatinib, apatinib and tivozanib, formed hydrogen bonds with the E885, C919 and D1046 amino acid residues of the VEGFR2 binding site, respectively. Two of the identified compounds (ZINC1033964 and ZINC114898570) showed hydrogen bond formation with all three of the aforementioned amino acid residues, while each of the other three selected compounds (ZINC1162830, ZINC6506329 and ZINC33268577) showed hydrogen bond formation with only two of these amino acid residues. Additionally, it is known that the nonbonded interactions with H1026 and Leu840 are of high importance for the binding of small molecules into the active site of VEGFR-2 protein. Such interactions were observed in the cases of all the selected compounds, with the exception of ZINC114898570, that did not show interactions with H1026. Moreover, the selected compounds showed additional nonbonded interactions with multiple amino acid residues in the binding pocket of the proteins, including V867, I888, V889, V898, C1024 and I1044, which could positively affect the selectivity of the molecules toward the VEGFR2 protein. Therefore, these compounds are of great interest for further research as potential inhibitors of the VEGFR2 protein.

## Supplementary Materials

The following are available online at https://www.mdpi.com/article/10.3390/life11101070/s1, Table S1: List of compounds with higher docking scores in comparison to tivozanib. Table S2: 2D structures of the 53 identified compounds as the result of the virtual screening and tivozanib.
